# Application of Taxonomic Modeling to Microbiota Data Mining for Detection of Helminth Infection in Global Populations

**DOI:** 10.3390/data1030019

**Published:** 2016-12-13

**Authors:** Mahbaneh Eshaghzadeh Torbati, Makedonka Mitreva, Vanathi Gopalakrishnan

**Affiliations:** 1 Department of Computer Science, University of Pittsburgh, 6135 Sennott Square, 210 S Bouquet St, Pittsburgh, PA 15260-9161, USA; 2 Department of Medicine, Washington University School of Medicine, 660 S Euclid Ave, St. Louis, MO 63110, USA; 3 Department of Biomedical Informatics, University of Pittsburgh, 5607 Baum Boulevard, Suite 500, Pittsburgh, PA 15206-3701

**Keywords:** helminth infection, microbiota, 16S rRNA gene, taxonomic tree, classification, SMART-scan method, transfer learning

## Abstract

Human microbiome data from genomic sequencing technologies is fast accumulating, giving us insights into bacterial taxa that contribute to health and disease. The predictive modeling of such microbiota count data for the classification of human infection from parasitic worms, such as helminths, can help in the detection and management across global populations. Real-world datasets of microbiome experiments are typically sparse, containing hundreds of measurements for bacterial species, of which only a few are detected in the bio-specimens that are analyzed. This feature of microbiome data produces the challenge of needing more observations for accurate predictive modeling and has been dealt with previously, using different methods of feature reduction. To our knowledge, integrative methods, such as transfer learning, have not yet been explored in the microbiome domain as a way to deal with data sparsity by incorporating knowledge of different but related datasets. One way of incorporating this knowledge is by using a meaningful mapping among features of these datasets. In this paper, we claim that this mapping would exist among members of each individual cluster, grouped based on phylogenetic dependency among taxa and their association to the phenotype. We validate our claim by showing that models incorporating associations in such a grouped feature space result in no performance deterioration for the given classification task. In this paper, we test our hypothesis by using classification models that detect helminth infection in microbiota of human fecal samples obtained from Indonesia and Liberia countries. In our experiments, we first learn binary classifiers for helminth infection detection by using Naive Bayes, Support Vector Machines, Multilayer Perceptrons, and Random Forest methods. In the next step, we add taxonomic modeling by using the SMART-scan module to group the data, and learn classifiers using the same four methods, to test the validity of the achieved groupings. We observed a 6% to 23% and 7% to 26% performance improvement based on the Area Under the receiver operating characteristic (ROC) Curve (AUC) and Balanced Accuracy (Bacc) measures, respectively, over 10 runs of 10-fold cross-validation. These results show that using phylogenetic dependency for grouping our microbiota data actually results in a noticeable improvement in classification performance for helminth infection detection. These promising results from this feasibility study demonstrate that methods such as SMART-scan can be utilized in the future for knowledge transfer from different but related microbiome datasets by phylogenetically-related functional mapping, to enable novel integrative biomarker discovery.

## 1. Introduction

The World Health Organization estimates that approximately two billion people are infected with parasitic helminths worldwide [1]. Helminths are parasitic worms that feed on a living host to gain nourishment and protection, while causing poor nutrient absorption, weakness and disease in the host. Soil-transmitted helminths cause most of the helminth infections and if an infected person or animal has defecated on soil, helminth eggs, that are present in their feces, contaminate the soil. People in developing countries, especially children who play in such contaminated soil, easily acquire helminth infection when worms, in the infective life cycle stage, penetrate human skin or are swallowed accidentally. These worms can cause several diseases, including severe pathologies of the intestine, liver, lungs, or brain. The use of 16S rRNA sequencing technologies to determine microbial community structure (taxa detected and their abundance; non-negative integers) of bacterial strains from such fecal specimens is becoming a popular method to study the impact of bacteria on human health and disease [2,3]. Even though next generation sequencing platforms have advanced microbiota profiling, several challenges remain. These include (1) sparseness of samples from which the microbiota data are acquired due to resource constraints, i.e., low coverage [4]; (2) sparseness of data since many species are not detected, resulting in zero counts, i.e., low abundant taxa due to primer bias [5]; and (3) the relatively recent development of automated methodologies for integrative analysis of human microbiota data, as it is a rapidly evolving and upcoming field of research, i.e., use of suboptimal tools [6].

For studying the association between microbiota features and host phenotypes to deal with challenges of this domain, many statistical methods have been proposed which use sequence counts as microbiota data. These statistical analysis methods can be categorized into several approaches: (a) simple univariate methods and tests of diversity; (b) multivariate methods; and (c) model-selection methods [7]. Univariate approaches ignore the multivariate and correlated nature of the taxa count and test each feature individually. These methods are considered to be effective when few taxa have strong effects on the host phenotype. White et al. use a non-parametric t-test based on permutation or a Fisher’s exact test for the sparse data to recognize organisms whose differential abundance is correlated with disease [8]. In linear discriminant analysis (LDA) effect size (LEfSe), a univariate test (Welch’s tor Wilcoxon rank test) is used to assess the significance of each feature [9]. First, LEFSe uses the Kruskal–Wallis (KW) ranksum test to estimate whether or not values in different classes are differentially distributed. Then, features violating the null hypothesis in the KW test are further analyzed using the paired Wilcoxon test between subclasses of data to test whether all pairwise comparisons between subclasses of classes are significantly compatible with the class level trend. Finally, the Latent Dirichlet Allocation (LDA) is used for ranking the relevant features. The resulting features are ranked according to the effect size with which they distinguish classes.

Multivariate methods calculate the association of the vector of multiple variables. In [10,11], different methods have been proposed to model variable abundances as drawn from a Dirichlet-multinomial, or a mixture of Dirichlet multinomials. Various non-parametric multivariate tests are based on pairwise distance measures that can be tree-unaware or tree-aware. Based on pairwise distances, various non-parametric multivariate tests can be conducted, such as the Mantel test and multivariate analysis of variance (MANOVA) [12–14]. One example of a tree-aware method is UniFrac, which calculates distances between pairs of microbiota samples in the phylogenetic tree [15].

A final category of methods for analyzing association between taxa abundances and host phenotypes consists of regression-based variable-selection methods, such as methods of the Partial Least Squares (PLS) family. PLS is a regression model that tries to find the direction in the observable variables (taxa) space that leads to the maximum multidimensional variance direction in the predicted variables (phenotype) space by projecting both types of variables to a new space [16]. Even though PLS is originally designed for regression problems, it performs well for classification problems too [17,18]. Sparse PLS (sPLS) was proposed in [19,20] to add Lasso penalization combined with Singular Value Decomposition (SVD) computation into PLS in order to handle sparse data. By considering predicted variables as another dataset of observable variables, this method was initially designed to identify the subsets of correlated variables of two different types in a way that each step consists of both variable selection and modeling procedures [21]. Sparse PLS Discriminant Analysis (sPLS-DA) is another method of the PLS-family which is an extension of sPLS to a supervised classification framework [22]. For gaining classification ability, the predicted variables can be coded with dummy variables to indicate the class of each observation. The class of a test observation can be selected based on the distance measures such as, maximum distance, centroid, or Mahalanobis. Another framework that extends sPLS-DA for metagenomic discovery is called mixMC, which identifies significant discriminative observable variables for preparing interpretable outputs. The mixMC framework uses Principal Component Analysis (PCA) to visualize diversity patterns and sPLS-DA to find indicator species or determinant microbiota members discriminating habitats or body sites [23].

While diversity tests and multivariate methods are usually used to determine whether there is an association between a set of taxa and the phenotype, univariate tests and model-selection methods extract these sets of features if the association exists. However, most of these methods model taxa as independent variables and ignore the phylogenetic relationship among them. Moreover, the regression-based variable-selection models, such as the PLS-family methods, assume multicollinearity between data. These methods also project the variables into a new space in order to satisfy their goals, such as predicting variables, dealing with data sparsity, or expanding visualization power. However, projecting features reduces the result interpretation, since the new features can only be approximately associated with the original feature set. Thus, interpretation and visualization in these methods are just limited to depicting samples in a two-dimensional space of projected components which allows manual subjective approximation of the association between the observable and predicted variables.

A recent innovative method, SMART-scan, for analyzing human microbiota data incorporates a taxonomic tree to increase modeling precision [7]. This method deals with the shortcomings of previous workflows in the way that it uses the phylogenetic dependency among taxa to not only eliminate the uninformative variables, but also to group the functionally similar variables into separate sets of grouped data with underlying biological connectivity. The main advantage of this method is its highly interpretable results, as no projection method is used and the new groups contain the original taxa which can be easily visualized in the phylogenetic tree. The SMART-scan algorithm was tested on a simulated dataset and the gut microbiota of vervet monkeys under two different diets: normal and fatty diets. For the simulated data, the authors compare SMART-scan to other statistical methods: Single-Variable analysis (SVA), stepwise regression, LASSO used in [24,25], and classification and regression trees (CART) [26]. The authors showed that SMART-scan has higher power than other statistical methods when there are group effects and its power drops if there is no group effect. For the vervet monkeys, they wanted to investigate the effects of a fatty diet on microbiota composition. They showed that the application of SMART-scan can reveal important features associated with groups and result in relevant parts of the phylogenetic tree associated with microbiota changes in response to diet, which otherwise would not have been detected by other non-tree aware approaches.

In this paper, we apply the SMART-scan algorithm to the analysis of two microbiota datasets acquired from 16s rRNA sequencing of stool specimens from global human populations with and without helminth infection. We believe that our paper is the first application of this automated phylogenetic clustering method to human microbiota datasets. Our long-term goal is to present an integrative biomarker discovery workflow that extracts robust, generalizable, and interpretable propositional rule patterns, showing the association between taxa and phenotypes. Previous workflows suffer from subjective interpretation and strong assumption of phylogenetic independency among taxa. Our workflow is designed to facilitate knowledge transfer from different but related microbiome datasets by phylogenetically-related functional mapping. In [27], functional mapping was introduced for relating different sparse biomedical datasets obtained for the same classification task, in order to effectively transfer information for integrative biomarker discovery. Therein, clusters of functionally-similar features are extracted from gene expression knowledge bases, and used to map variables between the related datasets. Herein, our goal is to automatically extract such functional mappings for microbiota data. We have this general hypothesis that “applying SMART-scan to multiple microbiota datasets that share phylogenetically dependent features would not decrease the classification performance for helminth infection detection over using just a single dataset, while allowing for grouping of taxa and phenotype associations at a more general and robust level”. We test this hypothesis using two datasets of helminth microbiota as starting points, and then by learning classifiers with and without SMART-scan groupings to observe the changes in predictive performance measures. The rest of this paper is organized to present the methods, datasets, experiments, and results used to demonstrate the feasibility of this hypothesis. This demonstration of feasibility is the first step towards the longer-term goal of enhancing our in-house developed machine learning methods to transfer learning of the classification rules to the application of microbiota data via the use of these phylogenetic groupings.

## 2. Materials and Methods

### 2.1. Microbiota Data

The input data that we use for this feasibility study is the microbiota count data obtained from 16s rRNA sequencing of stool samples from helminth-infected and helminth-non-infected individuals in two countries: Indonesia and Liberia. Single infections can be caused by different types of helminth worms (hookworm, ascaris or whipworm) and some samples are from multiple infections where a combination of different types of helminth worms are involved. Samples of each country are collected as a separate dataset referred to by the country name. The V1–V3 hypervariable regions of the 16S rRNA gene were amplified by PCR, and the PCR products from the Liberia samples were purified and sequenced on the MiSeq Genome Sequencer (average 24,000 reads per sample) and the Indonesia samples on the Genome Sequencer Titanium FLX (average 6000 reads per sample). Analytical processing of the data, and downstream analysis was performed as previously described [28]. The read counts per taxa obtained with the V1–V3 16S rRNA primers are provided as supplementary tables: Indonesia.csv (Table S5) and Liberia.csv (Table S6). A list of specific taxa and the complete cohort information will be published as a separate study and the sample ID will enable one to cross-link the information among the studies. In that study, they have the same primers for both platforms and the only difference is the depth of coverage, which means Illumina would detect some taxa with lower abundance vs the 454 platform. They did also compare the platforms for around 30 samples sequenced on both and 92% of the data detected with the 454 platform (the lower coverage platform) were also detected with Illumina.

Table 1 provides the distribution of the number of samples, the distribution of the infected (Helminth-infected; qPCR detection at a Threshold Cycle (CT) value of <28) and non-infected (Helminth-non-infected; no qPCR detection) classes, and the distribution of single-infected and multi-infected samples, with 702 bacterial taxa variables for Indonesia and Liberia datasets.

### 2.2. Methodology

Classification is the problem of assigning new observations to predefined categories [29]. In the classification problems, the observations are represented by a set of features. A feature is an individual measurable property of an observation. For solving the classification problems, machine learning or statistical models, called classifiers, are mainly used. These models learn the patterns identifying categories of the problem on the basis of a training set of data containing observations whose category membership is known. Models can then be used to classify new observations based on the patterns learned during the training phase.

In this study, we are trying to recognize whether or not an individual is infected by a parasitic worm. We have this hypothesis that features of microbial communities are associated with the host microbiota phenotype in such a way that we can extract some patterns from the set of detected taxa representing helminth infection. One way of checking this hypothesis is designing the task as a classification problem of categorizing observations into two categories of Helminth-infected versus Helminth-non-infected, in which the observations are represented by the detected taxa and their abundances, along with the phylogenetic tree structure. For directing the classification towards focusing on the dominancy of detected taxa, we utilize the relative abundance of taxa as the feature representation. While the majority of the Ribosomal Database Project (RDP) classifications are at a genus level [30], this is not always the case, therefore we will use the *taxa* as a broader classification term.

Using microbiota data causes two main challenges: sparseness of observation due to resource constraints and sparseness of taxa since many taxa are not detected in observations. Moreover, an actual dataset from microbiota experiments usually contains hundreds of taxa variables. For instance, for the helminth data, we have 702 taxa for just 90 and 74 observations of Indonesia and Liberia, respectively. The microbiota data presents two main problems for classification. First, as the dimension of the data increases, the number of observations needed for model training and consequently the study costs increase too. Second, if we even ignored the need for more training observations, we would also encounter other probable problems, such as the curse of dimensionality [31], or complicated models in need of longer training time [32]. Feature reduction is one solution for dealing with this problem in a way that tries to exclude redundant or uninformative features [33]. One other solution would be using integrative methods, such as transfer learning to feed models with different but related knowledge. As a way to incorporate this knowledge, a transfer learning approach using the functional mapping method has been proposed in [27]. We have this hypothesis that by applying SMART-scan on phylogenetically dependent microbiota datasets, we would extract clusters representing functional mapping to detect the association between taxa and phenotypes and also deal with data sparsity of microbiota data. To prove our hypothesis, we should just show that applying detection models to a microbiota dataset consists of new features (clusters) that do not result in performance deterioration.

**Figure 1:**
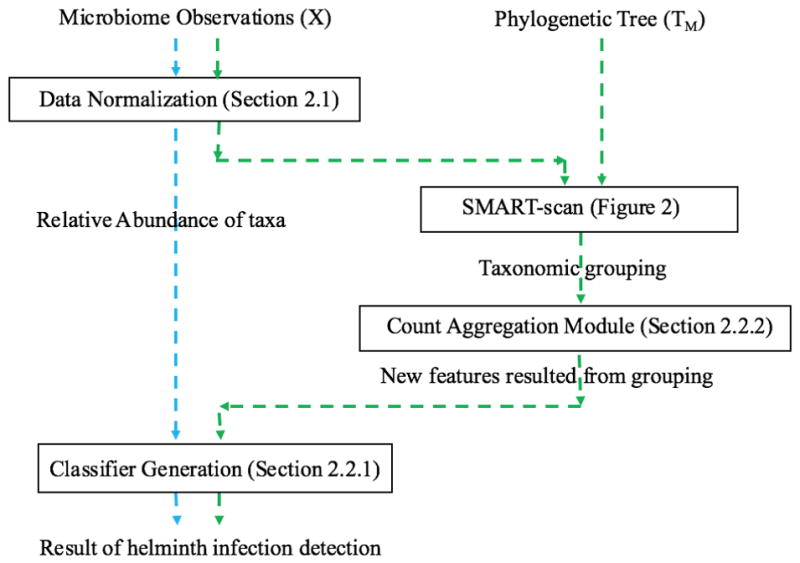
Flowchart of baseline and taxonomic models. The phylogenetic tree is one of the outputs of Ribosomal Database Project (RDP) [30].

Figure 1 depicts the experimental design for testing our specific hypothesis for helminth data that: “there exists a phylogenetic dependency among two helminth-associated microbiota datasets which can be used by SMART-scan to extract clusters representing functional mapping to identify the association between taxa and phenotypes; and that use of this grouped data does not decrease the classification performance for infection detection over that of the ungrouped data models”. In this regard, we designed both baseline (from ungrouped data) and taxonomic (from grouped data) models, depicted in blue and green dashed paths in Figure 1, respectively. Baseline is a classification model that uses classifier generation explained in Section 2.2.1 below. The taxonomic model is the baseline model with an additional SMART-scan module, as explained in Section 2.2.2. The taxonomic tree can be easily extracted from the separate study publishing the helminth datasets. In the following subsections, we discuss both the set of classifiers selected for the system, and the taxonomic model that uses the SMART-scan algorithm in more detail.

#### 2.2.1. Classifier Generation

We use a diverse set of four popular machine learning classifiers to first find an acceptable model for helminth detection and then study how SMART-scan can influence different classification models. For this purpose, we selected naive Bayes, support vector machines (SVMs), multilayer perceptrons (MLPs), and random forest classifiers. For the convenience of discussion, let us define *T_M_* as the taxonomic tree of the set of *M taxa*, *X* as a *N* by *M* matrix of *N* microbiota observations, *X* = [*X*_1_, *X*_2_, …, *X_N_* ], and *Y* as the phenotype vector of observations with *K* possible values.

##### Naive Bayes (NB)

The naive Bayes is a probabilistic model for estimating conditional distribution over the class variable, given a new observation [34]. Based on this model, we can assign the phenotype value *k* to the new helminth observations, *X_i_* = (*x*_1_, *x*_2_, *x*_3_, …, *x_M_*), as follows:

(1) $$k=\underset{k\in \{1,\ldots ,K\}}{\text{argmax}}\;p(Y_{k}|X_{i})$$

By consecutively applying Bayesian rule, ignoring *P*(*X_i_*) by considering it as a constant value for each observation, and assuming strong independency among features, we can rewrite the above equation as follows:

(2) $$k=\underset{k\in \{1,\ldots ,K\}}{\text{argmax}}\;P(Y_{k})\prod_{j=1}^{M}p(x_{j}|Y_{k})$$

Naive Bayes would result in high classification performance on independent data. Since SMART-scan aims at grouping feature sets into independent clusters, the combination of the SMART-scan and the naive Bayes classifier would result in a system with high performance.

##### Support Vector Machines (SVMs)

In the SVMs model, observations are represented as points in a multidimensional space of features [35]. SVMs produce a discriminative model by learning that the hyper-plane separates observations of different categories in a way that makes the inter-category distance as large as possible. A new observation is classified based on the learned hyper-plane separator. Large numbers of features may reduce the performance of discriminative models. Since SMART-scan aims to eliminate the inefficient and redundant features, the combination of the SMART-scan and any discriminative model (such as SVMs) may result in a high-performing system.

##### Multilayer Perceptrons (MLP)

The MLP is a discriminative model, a network of simple neurons [36]. The neurons are structured in one input layer at the beginning, at least one hidden layer in the middle, and one output layer at the end of the network. The number of hidden layers and the number of neurons in each of these layers depend on the network design, while the number of neurons in the input and output layers depends on the number of features and classes, respectively. The neurons of all layers, except the output layer, are fully connected to the neurons of the next layer by weighted edges. These weights are the parameters learned during the model training phase. Each neuron has an activation function, a mathematical function accepts weights and input data from the previous layer and feeds the neurons of the next layer by the function’s output. By giving the new observation to the input layer of the trained network, we will have the predicted class as the output of the last layer. SMART-scan may improve the performance and computation time of the MLP by feature reduction. By selecting neural network models as classifiers, we may suffer from considerable computation time and a low interpretation level of the model, even though it may result in high performance. We use MLP in our experiments to test whether or not we can substitute it with simpler and faster models with comparable classification power.

##### Random Forests (RF)

Random forests is an ensemble learning method, i.e., it is a multitude of random decision trees for obtaining better predictive performance and dealing with the overfitting problem by selecting the mode of the class labels predicted by the decision trees [37]. Decision tree is a discriminative model that results in a trained classification tree in which the leaves are the classes, and the interior nodes of each level are possible values of the selected feature for that level [38]. In the train phase, for each level, the feature with the highest information gain (IG) is selected [39]. In the test phase, the class label of the leaf that matches the conjunctions of feature values of the new observation is selected. By using RF, we want to benefit from ensemble learning models while also testing whether or not selecting features based on another measure, IG, may result in a classification performance improvement.

#### 2.2.2. Taxonomic Modeling

The many possible ways of grouping detected bacterial taxa variables leads to a big hypothesis space to be searched exhaustively. One way to deal with this issue is the heuristic technique proposed in the artificial intelligence (AI) domain. When finding an optimal solution for a class of problems is impossible, impractical, or time-consuming, heuristic methods can be used for finding a satisfactory solution in a shorter time. Heuristics are usually mental shortcuts that help to ignore unlikely solutions. One example of using heuristics is in the A* method as one solution of “minimizing the total path cost” problem [40]. The goal of the SMART-scan is to efficiently put correlated taxa in distinct groups. The SMART-scan follows the heuristic that: *those taxa that share an evolutionary ancestry path in the phylogenetic tree are biologically related and are more likely to be in the same group*. The taxonomic modeling is a classification modeling with an additional SMART-scan module [7].

SMART-scan is an iterative algorithm of three main steps: model creation, model evaluation, and model selection. In each iteration, the splitting of a phylogenetic sub-tree into two sub-trees is evaluated. In the model creation step, all possible group models resulted from applying cut-points that split the sub-tree into two sub-trees are produced. Let us call the model before applying the splits, *Model*_0_. In the model evaluation step, all the produced models of previous step are evaluated based on Akaike information criterion (AIC) measure. By AIC, the information lost when a model is used to represent the process that generates data is estimated. Among the models of this step, the model with the smallest AIC is selected as the *Best-Model*. The model selection step checks whether or not the applied grouping results in model improvement. Thus, *Best-Model* is compared to *Model*_0_ based on their AIC measures and the model with the lower AIC is selected for the iteration. If the *Best-Model* is the selected model of the iteration, the sub-trees resulted from the split are tested for grouping in the future iterations. By the end of the algorithm, we can consider each of the resulted groups as a new feature valued by the aggregation of the group members’ values.

**Figure 2:**
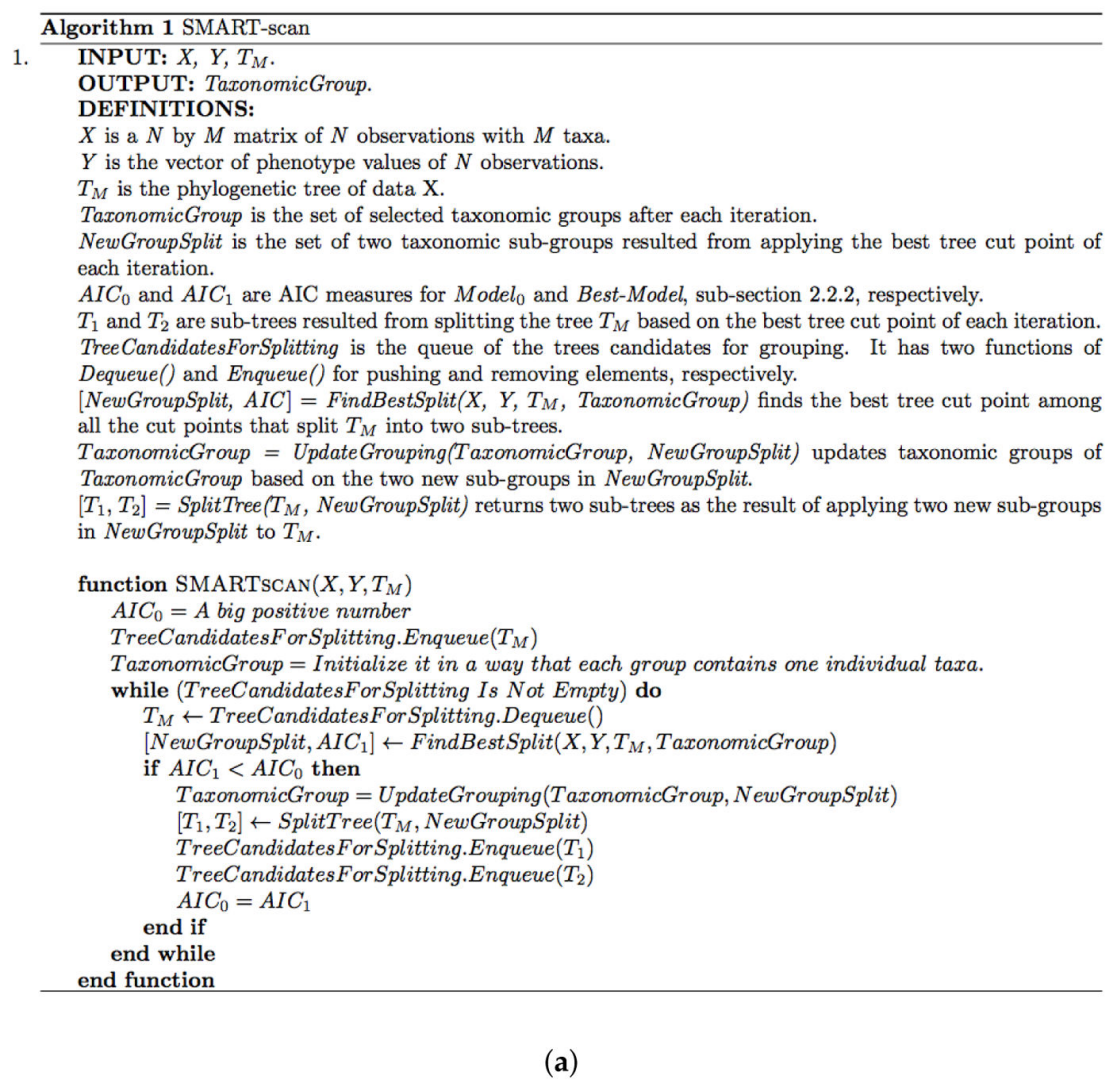

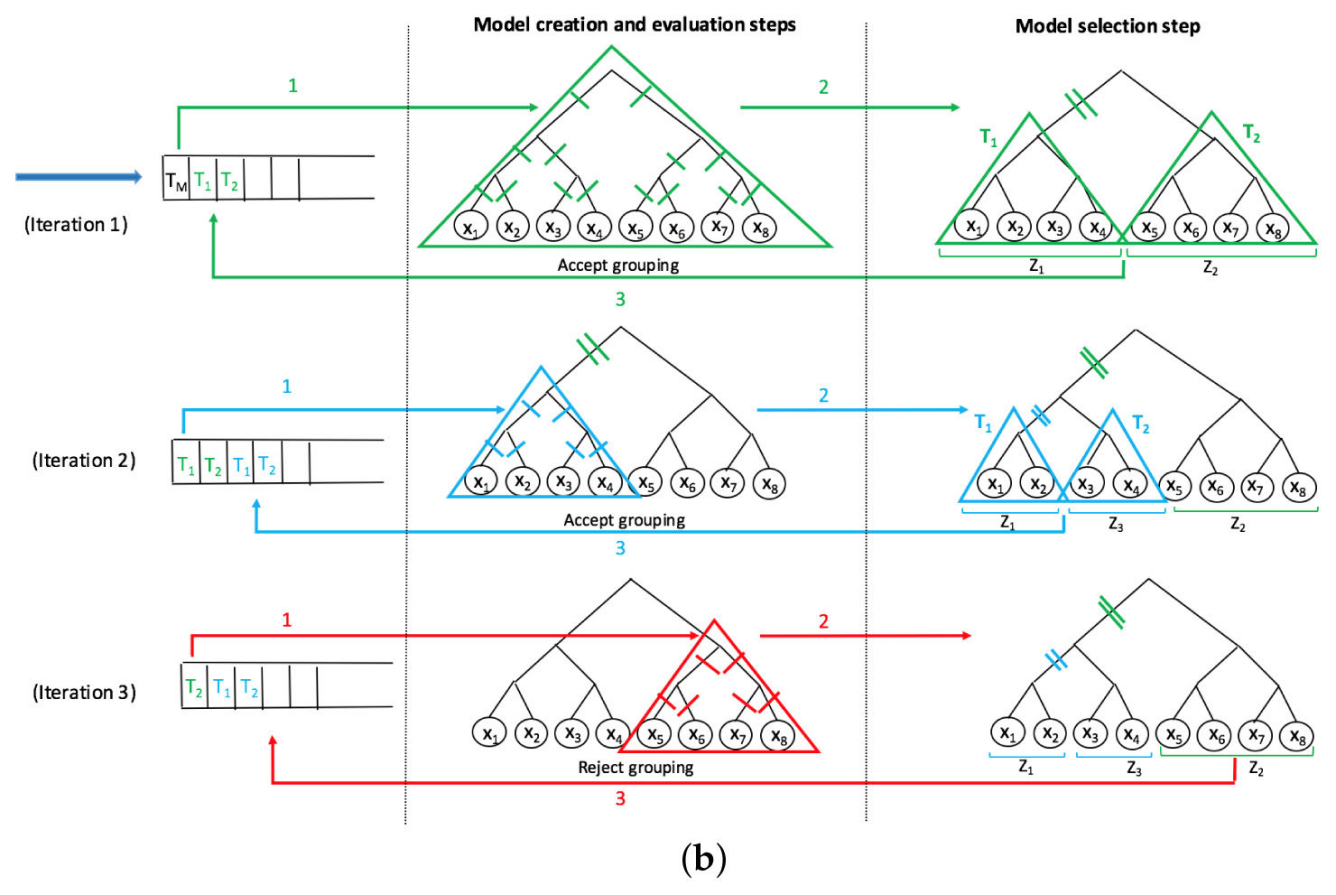
The pseudocode and first three iterations of the SMART-scan algorithm derived from the explanations provided in the main paper [7], and the R code provided by the authors of this paper. The derived psuedocode in (**a**) is meant to provide a computational interpretation and rapid lookup to enhance the extensions to the application of the SMART-scan method for automatic clustering of microbiota. The first three iterations of the SMART-scan algorithm in (**b**) are prepared based on the pseudocode in (**a**). In the first column, the sub-tree candidate for grouping is extracted from *TreeCandidatesForSplitting*. In the second column, the sub-tree is enclosed by a triangle in the phylogenetic tree; all the possible cut points of the sub-tree are marked by lines cutting edges, and the taxa are named as *x_i_ s*. In the third column, the selected cut points are depicted by double lines, the selected grouping of taxa are named as *Z_i_ s*, and the new splitted sub-trees, named as *T*_1_ and *T*_2_, are pushed into *TreeCandidatesForSplitting*.

Figure 2 depicts the pseudocode and visualization of the first three iterations of the SMART-scan algorithm. In the pseudocode shown in Figure 2a, two main data structures are used: *TreeCandidatesForSplitting* and *TaxonomicGroup. TreeCandidatesForSplitting* is a queue that contains the tree candidates for splitting (grouping), initially filled with *T_M_*; and *TaxonomicGroup* is the list of groupings, initially filled with the groups each containing one individual taxa. The three steps of the algorithm are covered in the *While* loop in a way that the model creation and evaluation steps have been implemented by *FindBestSplit()* function and the model selection step and the data structure updates have been implemented by the *If* statement and its inner instructions. In the visualization shown in Figure 1b, the first column shows the *Enqueue* and *Dequeue* functions applied to the *TreeCandidatesForSplitting* queue. The second column shows the sub-tree candidate for grouping and all its possible cut points. The third column shows all the tree cut points and the groups of taxa selected by the algorithm so far. The order of the actions in each iteration has been shown by numbered arrows. As it is shown in Figure 2b, the algorithm accepts groupings of the first two iterations, while it rejects the suggested grouping of the third iteration.

## 3. Results and Discussion

### 3.1. Experimental Evaluation

The evaluation of both models over Indonesia, Liberia, and Combined helminth datasets, using 10 runs of 10-fold cross-validation based on the percentage of the area under the ROC curve (AUC), are reported in Tables 2–4, respectively. We also have the same evaluation based on the Sensitivity (Sen), Specificity (Spec), and Balanced Accuracy (Bacc) measures in Tables 5–7. The Bacc measure reports the average of sensitivity and specificity for the detection of infection, thereby capturing directly the trade-offs between the two measures. The Combined dataset, is the combination of both Indonesia and Liberia datasets. We have this additional dataset as a way to directly increase the small numbers of observations, by a simple unioning of the samples for the set of common features among the two datasets. (It is to be noted herein that this simple unioning does not allow for many common features that may be related biologically through functional or phylogenetic dependencies to be utilized—which is our main motivation for transferring knowledge through functional mapping in the future.) For testing whether the achieved improvement by the taxonomic modeling is statistically significant, we have done both a non-parametric Wilcoxon paired-sample signed-rank test and paired-sample two-tailed t-test, with a significance level *α* = 0.05 over the 10 runs for each classifier. For our experiments, we used the Weka implemented version of the classifier generation with their default parameter set in this software [41]. The SMART-scan algorithm developers made the code publicly available [42], which allowed us to apply it and also evaluate it on our microbiota data.

### 3.2. Discussion

By using SMART-scan, we reduced the number of features (bacterial taxa) from 702 to 56, 53, and 62 for Indonesia, Liberia, and Combined datasets, respectively. Results in Tables 2 and 3 show statistically significant helminth detection AUC improvement achieved by using the taxonomic model over both Indonesia and Liberia datasets, except for the multilayer perceptron and random forest classifiers, highlighted in Table 3. The same improvement can also be observed from Table 4 and it shows that preparing the Combined dataset for dealing with the shortage of observations is an effective solution. The same improvement can also be seen in Tables 5–7 for Specificity, Sensitivity, and Balanced accuracy measures. The results of these tables not only validate our hypothesis that “applying SMART-scan to helminth data would result in groups of potentially functionally similar microbiota for detecting helminth infection”, but also show that this feature reduction achieved by SMART-scan is effective and results in statistically significant improvements for classification of helminth infection from such sparse microbiota datasets.

The observed patterns of performance improvement through the application of SMART-scan seem justifiable based on the theoretical foundations and assumptions of the four diverse classifiers chosen for testing our hypothesis in this feasibility study. Naive Bayes models, for example, are based on a strong feature independency assumption, and thereby we see that aggregated counts of detected bacterial taxa act as independent groups, and give rise to the statistically significant improvements shown above. For support vector machines and multilayer perceptrons, SMART-scan decreases the model complexity by shrinking the size of the feature set. In this way, it reduces the dimension of the feature space for support vector machines and decreases the number of neurons in the input layer for the multilayer perceptrons. Even in ensemble modeling algorithms such as Random Forests, which is a collection of decision trees with the goal of selecting the most informative set of features for the predefined classification task, SMART-scan improves overall performance on all the datasets as shown above. This consistent pattern of results seems to indicate that SMART-scan may be able to increase both speed and accuracy in classification of such data, via the use of a smaller set of aggregated features, each representative of phylogenetical correlations.

## 4. Conclusions

In this paper, we showed that taxonomic modeling, using a recently published method called SMART-scan, improves classification performance of real-world human microbiota datasets. Particularly, we tested the improvements in the AUC, Specificity, Sensitivity, and Balanced Accuracy performance over 10 runs of 10-fold cross-validation of application of SMART-scan to aggregate bacterial groups detected in two global populations with and without helminth infection. By showing the achieved improvement, we proved our hypothesis that “applying SMART-scan on phylogenetically dependent microbiota datasets would result in clusters representing functional mapping for to identify the association between taxa and phenotypes for our helminth datasets.” We also showed that by adding the SMART-scan algorithm to our models, we increased model performance by dealing with the problem of data sparsity.

We also tried four different classifiers in our experiments: naive Bayes, support vector machines, multilayer perceptrons, and random forests, with all leading to statistically significantly improved AUCs, specificity, sensitivity, and Balanced accuracy with the SMART-scan module. Moreover, we observe a much greater performance gain for those classifiers that have feature independency assumptions [naive Bayes] and those which map the classification problem to a feature space [support vector machines]. As our future work, we are aiming to study classification models adaptable to the SMART-scan groupings. Advancing the models would result in precisely defining the microbial ecology underlying helminth infections and determining whether microbiota assemblages, after deworming, resemble the healthy microbiota state.

## Figures and Tables

**Table 1:** Indonesia and Liberia datasets statistics.

Dataset ID	Number of Samples	Number of Taxa	Class Distribution (Helminth-Infected/Non-Infected)	Helminth-Infected Distribution (Single/Multi-Infected)	Type of Multi-Infection Worms
Indonesia	90	702	38/52	35/3	3 (ascaris + hookworm)
Liberia	74	702	23/51	19/4	3 (ascaris + hookworm) and 1 (ascaris + whipworm)

**Table 2:** Baseline and taxonomic model performance on detecting helminth infection over the Indonesia dataset, using 10 runs of 10-fold cross-validation. In this table and the two other following tables, columns are as follows (from left to right): the classifier generation method, the Area Under the ROC Curve (AUC) measures for the baseline and taxonomic models, the improvement achieved by the taxonomic model, and the *p*-value for t-test and Wilcoxon tests. Best results of columns’ Taxonomic Model AUC and Improvement are shown in bold font in the tables.

Classifier	Baseline AUC	Taxonomic Model AUC	Improvement	*p*-Value for [*t*-Test, Wilcoxon Test]
NB	0.69	**0.87**	0.18	[0.029, 0.039]
SVMs	0.61	**0.87**	**0.26**	[0.002, 0.000]
MLP	0.61	0.85	0.24	[0.004, 0.001]
RF	0.67	0.81	0.14	[0.039, 0.048]

**Table 3:** Baseline and taxonomic model (AUC) performance on detecting helminth infection over the Liberia dataset.

Classifier	Baseline AUC	Taxonomic Model AUC	Improvement	*p*-Value for [*t*-Test, Wilcoxon Test]
NB	0.71	**0.94**	0.23	[0.006, 0.002]
SVMs	0.52	0.82	**0.30**	[0.002, 0.000]

MLP	0.78	0.84	0.06	[0.180, 0.087]
RF	0.85	0.92	0.07	[0.062, 0.073]

**Table 4:** Baseline and taxonomic model (AUC) performance on detecting helminth infection over the *Combined* dataset.

Classifier	Baseline AUC	Taxonomic Model AUC	Improvement	*p*-Value for [*t*-Test, Wilcoxon Test]
NB	0.66	**0.89**	**0.23**	[0.002, 0.000]
SVMs	0.59	0.81	0.22	[0.002, 0.000]
MLP	0.75	0.87	0.12	[0.014, 0.008]
RF	0.72	0.85	0.13	[0.027, 0.024]

**Table 5:** Baseline and taxonomic model performance on detecting helminth infection over the Indonesia dataset, using 10 runs of 10-fold cross-validation. In this table and the two other following tables, columns are as follows (from left to right): the classifier generation, the Sensitivity/Specificity (Sen/Spec) for infection detection and the Balanced accuracy (Bacc) measures for the baseline and taxonomic models, the improvement achieved by the taxonomic model for Bacc, and the *p*-value for t-test and Wilcoxon tests. Best results of the columns’ Taxonomic Model Bacc and Improvement are depicted in bold font in the tables.

Classifier	Baseline Sen/Spec	Taxonomic Model Sen/Spec	Baseline Bacc	Taxonomic Model Bacc	Improvement Bacc	*p*-Value for [*t*-Test, Wilcoxon Test]
NB	0.58/0.67	0.86/0.77	0.62	0.82	0.20	[0.001, 0.004]
SVMs	0.26/0.96	0.78/0.96	0.61	**0.87**	**0.26**	[0.000, 0.002]
MLP	0.51/0.73	0.82/0.78	0.62	0.80	0.18	[0.030, 0.040]
RF	0.41/0.88	0.57/0.85	0.64	0.71	0.07	[0.006, 0.030]

**Table 6:** Baseline and taxonomic model (Sensitivity, Specificity, and Balanced accuracy) performance on detecting helminth infection over the Liberia dataset.

Classifier	Baseline Sen/Spec	Taxonomic Model Sen/Spec	Baseline Bacc	Taxonomic Model Bacc	Improvement Bacc	*p*-Value for [*t*-Test, Wilcoxon Test]
NB	0.6/0.72	0.88/0.90	0.66	**0.89**	**0.23**	[0.020, 0.040]
SVMs	0.05/1.0	0.72/0.92	0.75	0.82	0.07	[0.000, 0.002]
MLP	0.63/0.82	0.68/0.92	0.72	0.80	0.08	[0.001, 0.002]
RF	0.18/0.98	0.52/0.98	0.58	0.75	0.17	[0.030, 0.040]

**Table 7:** Baseline and taxonomic model (Sensitivity, Specificity, and Balanced accuracy) performance on detecting helminth infection over the *Combined* dataset.

Classifier	Baseline Sen/Spec	Taxonomic Model Sen/Spec	Baseline Bacc	Taxonomic Model Bacc	Improvement Bacc	*p*-Value for [*t*-Test, Wilcoxon Test]
NB	0.59/0.70	0.87/0.79	0.64	0.83	**0.19**	[0.001, 0.002]
SVMs	0.21/0.97	0.67/0.88	0.59	0.77	0.18	[0.002, 0.006]
MLP	0.68/0.82	0.77/0.80	0.75	**0.87**	0.12	[0.036, 0.039]
RF	0.29/0.94	0.61/0.91	0.61	0.76	0.15	[0.001, 0.004]

## References

[1] World Health Organization (2004). Estimated Incidence, Prevalence and TB Mortality.

[2] Mendes-Soares H, Krishnan V, Settles ML, Ravel J, Brown CJ, Forney LJ (2015). Fine-scale analysis of 16S rRNA sequences reveals a high level of taxonomic diversity among vaginal *Atopobium* spp. Pathog Dis.

[3] Nistal E, Caminero A, Herrán AR, Pérez Andres J, Vivas S, Ruiz de Morales JM, Sáenz de Miera LE, Casqueiro J (2016). Study of duodenal bacterial communities by 16s rrna gene analysis in adults with active celiac disease versus non-celiac disease controls. J Appl Microbiol.

[4] Wendl MC, Kota K, Weinstock GM, Mitreva M (2013). Coverage theories for metagenomic DNA sequencing based on a generalization of Stevens’ theorem. J Math Biol.

[5] Jumpstart Consortium Human Microbiome Project Data Generation Working Group (2012). Evaluation of 16S rDNA-based community profiling for human microbiome research. PLoS ONE.

[6] Hill TC, Walsh KA, Harris JA, Moffett BF (2003). Using ecological diversity measures with bacterial communities. FEMS Microbiol Ecol.

[7] Zhang Q, Abel H, Wells A, Lenzini P, Gomez F, Province MA, Templeton AA, Weinstock GM, Salzman NH, Borecki IB (2015). Selection of models for the analysis of risk-factor trees: Leveraging biological knowledge to mine large sets of risk factors with application to microbiome data. Bioinformatics.

[8] White JR (2009). Statistical methods for detecting differentially abundant features in clinical metagenomic samples. PLoS Comput Biol.

[9] Segata N, Izard J, Waldron L, Gevers D, Miropolsky L, Garrett WS, Huttenhower C (2011). Metagenomic biomarker discovery and explanation. Genome Biol.

[10] Holmes I, Harris K, Quince C (2012). Dirichlet multinomial mixtures: Generative models for microbial metagenomics. PLoS ONE.

[11] La Rosa PS, Brooks JP, Deych E, Boone EL, Edwards DJ, Wang Q, Sodergren E, Weinstock G, Shannon WD (2012). Hypothesis testing and power calculations for taxonomic-based human microbiome data. PLoS ONE.

[12] Anderson MJ (2001). A new method for nonparametric multivariate analysis of variance. Austral Ecol.

[13] Chen J, Bittinger K, Charlson ES, Hoffmann C, Lewis J, Wu GD, Collman RG, Bushman FD, Li H (2012). Associating microbiome composition with environmental covariates using generalized UniFrac distances. Bioinformatics.

[14] Mantel N (1976). The detection of disease clustering and a generalized regression approach. Cancer Res.

[15] Lozupone C, Knight R (2005). UniFrac: A new phylogenetic method for comparing microbial communities. Appl Environ Microbiol.

[16] Tobias RD An introduction to partial least squares regression.

[17] Barker M, Rayens W (2003). Partial least squares for discrimination. J Chemom.

[18] Nguyen DV, Rocke DM (2002). Tumor classification by partial least squares using microarray gene expression data. Bioinformatics.

[19] Lê Cao KA, Rossouw D, Robert-Granié C, Besse P (2008). A sparse PLS for variable selection when integrating omics data. Stat Appl Genet Mol Biol.

[20] Lê Cao KA, Martin PG, Robert-Granié C, Besse P (2009). Sparse canonical methods for biological data integration: Application to a cross-platform study. BMC Bioinform.

[21] Mahana D, Trent CM, Kurtz ZD, Bokulich NA, Battaglia T, Chung J, Müller CL, Li H, Bonneau RA, Blaser MJ (2011). Antibiotic perturbation of the murine gut microbiome enhances the adiposity, insulin resistance, and liver disease associated with high-fat diet. Genome Med.

[22] Lê Cao KA, Boitard S, Besse P (2011). Sparse PLS discriminant analysis: Biologically relevant feature selection and graphical displays for multiclass problems. BMC Bioinform.

[23] Lê Cao KA, Costello ME, Lakis VA, Bartolo F, Chua XY, Brazeilles R, Rondeau P (2016). mixMC: A multivariate statistical framework to gain insight into Microbial Communities. bioRxiv.

[24] Sun Y, Cai Y, Mai V, Farmerie W, Yu F, Li J, Goodison S (2011). Advanced computational algorithms for microbial community analysis using massive 16S rRNA sequence data. Nucleic Acids Res.

[25] Tibshirani R (2011). Regression shrinkage and selection via the lasso: A retrospective. J R Stat Soc Ser B (Stat Methodol).

[26] Loh WY (2011). Classification and regression trees. Wiley Interdiscip Rev Data Min Know Dis.

[27] Ogoe HA, Visweswaran S, Lu X, Gopalakrishnan V (2015). Knowledge transfer via classification rules using functional mapping for integrative modeling of gene expression data. BMC Bioinform.

[28] Ordiz MI, May TD, Mihindukulasuriya K, Martin J, Crowley J, Tarr PI, Ryan K, Mortimer E, Gopalsamy G, Maleta K (2015). The effect of dietary resistant starch type 2 on the microbiota and markers of gut inflammation in rural Malawi children. Microbiome.

[29] Alpaydin E, Dietterich T, Bishop C, Heckerman D, Jordan M, Kearns M (2010). Supervised Learning. Introduction to Machine Learning.

[30] Cole JR, Wang Q, Fish JA, Chai B, McGarrell DM, Sun Y, Brown CT, Porras-Alfaro A, Kuske CR, Tiedje JM (2013). Ribosomal Database Project: Data and tools for high throughput rRNA analysis. Nucleic Acids Res.

[31] Bellman RE (1957). Dynamic Programming.

[32] Bermingham ML, Pong-Wong R, Spiliopoulou A, Hayward C, Rudan I, Campbell H, Wright AF, Wilson JF, Agakov F, Navarro P (2012). Application of high-dimensional feature selection: Evaluation for genomic prediction in man. Sci Rep.

[33] Roweis ST, Saul LK (2000). Nonlinear dimensionality reduction by locally linear embedding. Science.

[34] Rish I (2001). An empirical study of the naive Bayes classifier. IJCAI.

[35] Burges CJ (1998). A tutorial on support vector machines for pattern recognition. Data Min Know Dis.

[36] Panchal G, Ganatra A, Kosta YP, Panchal D (2011). Behaviour analysis of multilayer perceptrons with multiple hidden neurons and hidden layers. Int J Comput Theory Eng.

[37] Breiman L (2001). Random forests. Mach Learn.

[38] Quinlan JR (1986). Induction of decision trees. Mach Learn.

[39] Kent JT (1983). Information gain and a general measure of correlation. Biometrika.

[40] Russell SJ, Norvig P, Canny JF, Malik JM, Edwards DD, Pompili M, Chavez S (1995). Informed Search Methods. Artificial Intelligence: A Modern Approach.

[41] Hall M, Frank E, Holmes G, Pfahringer B, Reutemann P, Witten IH (2009). The WEKA data mining software: An update. ACM SIGKDD Explor.

[42] Zhang Q (2015). Implemented code for SMARTscan.

